# Corporate financialization, financing constraints, and innovation efficiency—Empirical evidence based on listed Chinese pharmaceutical companies

**DOI:** 10.3389/fpubh.2023.1085148

**Published:** 2023-04-14

**Authors:** Jialin Zhu, Yaning Tang, Yanyi Wei, Su Wang, Yuwen Chen

**Affiliations:** ^1^School of Business Administration, Shenyang Pharmaceutical University, Shenyang, China; ^2^Drug Regulatory Research Base of NMPA, Research Institute of Drug Regulatory Science, Shenyang Pharmaceutical University, Shenyang, China

**Keywords:** corporate financialization, innovation efficiency, financing constraints, listed pharmaceutical companies, empirical analysis

## Abstract

The relationship between financialization and innovation has become a common focus of academic attention. This paper analyzes the influence of corporate financialization on innovation efficiency based on balanced panel data of listed Chinese pharmaceutical companies from 2015 to 2020. Also, it examines the relationship between corporate financialization and innovation efficiency under different levels of financing constraints and the moderating mechanisms that exist. The results of the study show that corporate financialization negatively affects innovation efficiency and that this effect has a lag; corporate financialization hurts innovation efficiency across the different regions and firm nature, with a less inhibiting effect for eastern firms and non-state-owned firms; further tests of the mechanism of action show that there is a non-linear negative relationship between corporate financialization and innovation efficiency. And the inhibition of corporate financialization on innovation efficiency decreases as the level of financing constraints rises. Based on the above findings, this study provides warnings and recommendations for pharmaceutical companies to finance their innovative activities through financialization.

## 1. Introduction

Pharmaceutical manufacturing is one of the high-tech industries reflecting the strength of the national economy and is closely related to people’s health levels (1). The impact of the Newcastle pneumonia epidemic has further stimulated innovation and development in the global pharmaceutical industry. The competitiveness of pharmaceutical manufacturing has become one of the most critical elements in evaluating a country’s overall strength (2). Innovation is essential for the continued growth of the pharmaceutical manufacturing industry. On the one hand, innovation is an essential factor in the development of the industry. Only by improving innovation efficiency can the pharmaceutical manufacturing industry seize the market. On the other hand, pharmaceutical manufacturing is a knowledge-intensive industry driven by innovation and is typically a research and development industry. Among all industries, pharmaceutical manufacturing has the highest investment in research and development (3). For example, according to the National Institutes of Health (NIH), from 2008 to 2015, the U.S. federal government invested an average of about $30 billion per year in pharmaceutical R&D, with $41.9 billion invested in 2020. According to China High-Tech Industry Statistical Yearbook, from 2007 to 2020, R&D expenditure in China’s pharmaceutical manufacturing industry rose from RMB 130 million to RMB 78.46 billion. The innovation process requires significant investment in R&D. Compared with developed countries, China’s investment in R&D is far from adequate, and its innovation results are not as good as those of developed countries. China’s pharmaceutical manufacturing industry has serious problems with “emphasis on imitation, not originality” and “quantity, not quality” (4), and lacks internationally recognized original drugs. The Chinese pharmaceutical industry is still facing problems such as a low level of innovation and an inadequate innovation system (5). These problems are related to the inefficient innovation of the pharmaceutical industry. Pharmaceutical manufacturing needs R & D investment, more need for innovation efficiency, and quality of output (6). Therefore, how to improve the innovation efficiency of the pharmaceutical manufacturing industry and explore the factors affecting the innovation efficiency of the pharmaceutical manufacturing industry is an increasingly popular topic in academia.

The changing external environment has forced countries to cope with severe economic stagnation in recent years. Many businesses face a shortage of funds and a decline in earnings for the real economy. Theoretically, a firm’s willingness to innovate depends not only on the benefits of innovation but is also constrained by the resources invested in innovation. When the investment in innovation is not sufficient and external financing is not available, managers often look to the financial markets to make up for the lack of R&D funds, motivated by the profit motive of capital. However, financial markets face severe instability problems.

The U.S. government data shows the financial, insurance, and real estate sectors ‘share of the U.S. GDP rose from 15 to 24%, outstripping manufacturing (7). This situation also occurred in China. Value added in China’s financial sector as a share of GDP continued to rise from 2007 to 2016 (8). Enterprises gradually change the traditional way of investment and will progressively shift funds to venture capital (9). The same problem exists in the pharmaceutical manufacturing industry. According to CSMAR, the stock of financial assets of listed pharmaceutical companies in China rose from 12.6 billion yuan to 150.2 billion yuan between 2010 and 2020. When the capital demand for research and development is high, and the innovation results are unstable, pharmaceutical managers tend to choose financial assets that yield high returns in a short time frame. Thus enterprise neglects the long-term growth of the enterprise, causing the resources of the company to flow into the financial sector and reducing the company’s investment in research and development (10). However, the output of innovation results requires the support of R&D funds. The allocation of financial assets has crowded out resources originally used for pharmaceutical innovation, resulting in a lack of initiative in technological innovation and R&D investment by pharmaceutical enterprises, which limits enterprises’ creative output and innovation efficiency. Financial investment, a fast and profitable way of financing, often crowds out R&D funds, making pharmaceutical companies inefficient at innovation. Therefore, it is worth exploring how to reduce the negative impact and increase innovation efficiency when financialized companies.

At present, most of the research only analyzes the effect of financialization on innovation input and output, and few have explored the mechanisms by which corporate financialization affects innovation efficiency. Therefore, this paper uses data from a sample of Chinese-listed pharmaceutical companies to investigate the influences of corporate financialization on the innovation efficiency in Chinese pharmaceutical companies and to delve into the heterogeneity of financing constraints and the mechanisms of effect. Compared with existing studies, the contributions of this paper are: first, there are few studies on the financial market investment of pharmaceutical enterprises. This paper explains the low investment efficiency of China’s pharmaceutical manufacturing industry from the perspective of entity financialization and provides investment and financing suggestions for the managers of pharmaceutical enterprises. Secondly, this paper attempts to expand the research scope of previous scholars and comprehensively considers the impact of financialization on enterprise innovation efficiency. Innovation efficiency reflects the effectiveness of innovation input and innovation output. Thirdly, this paper discusses the non-linear relationship between corporate financialization and innovation efficiency, brings financing constraints into the research framework, and explores its non-linear relationship and mechanism as a threshold variable and a moderator variable. The rest of this article is arranged as follows. In the part of “literature review and research hypothesis,” this paper reviews the previous literature and puts forward the hypothesis of this paper. The “materials and methods” section provides data sources, variables selection, and model settings. The “empirical analysis results and discussion” section gives the empirical results. The conclusion gives “conclusion and discussion, policy proposal, limitations and future work.”

## 2. Literature review and research hypothesis

### 2.1. Corporate financialization

It is now common for companies to make a financial investment in the trend of the profit motive of capital (10). There is no consensus on the definition of financialization Sawyer (11) pointed out that financialization is a significant investment in financial markets relative to the real economy. One of the micro-level manifestations of corporate financialization is the increasing share of profits from financial channels, with some enterprises even relying on them to maintain their essential profitability (12). Thus, the company neglects its original main business.

In theory, corporate financialization has a two-way effect on economic growth. On the one hand, companies can stimulate their short-term performance through financial investment practices, resulting in short-term gains (13). However, financialization is often prone to speculative behavior, which has negative economic consequences. Excessive financialization tends to make companies neglect their primary business, reduce productivity, and harm their development (14). The degree of corporate financialization significantly relates to whether enterprises tend to have speculative motives or savings motives for financial investment. The above study is based on a linear perspective to explore. Huang et al. (15) argued that financialization has a U-shaped, non-linear relationship with the economy.

In addition, Wang et al. (16) showed that corporate financialization heterogeneity affects firms’ total factor productivity. At the same time and conversely, the financialization of the economy can cause the alienation of financial investment behavior (17). Although financialization can crowd out other investments in the business to some extent, Bloom et al. (18) showed that financial investment behavior by firms can hedge against price and exchange rate fluctuations, alleviate financing difficulties, and promote physical investment.

### 2.2. Corporate financialization and innovation

As an essential dimension of financial development, financialization is also important for the innovative development of enterprises. On the one hand, the financial investment of enterprises has become the main investment direction of non-financial companies. When holding financial assets is to “save”， financial investment can cope with the risk of research and development and maintain the continuity of innovation investment (19).

On the other hand, financial assets are held by firms for “speculative purposes,” driven more by their yield differentials and based on this motivation. Financial investment behavior can encroach on the capital investment of firms’ innovation activities. González et al. (3) have shown that the financial asset allocation of enterprises significantly reduces the R&D investment of enterprises. Innovation activities have the characteristics of a long cycle and high R&D, which require a large amount of capital injection. The increasing financial investment will crowd out R&D and innovation investment, slow down the improvement of enterprise technology level, innovation quality, and production capacity, reduce innovation achievements and inhibit the improvement of enterprise production efficiency (20). Similarly, Akkemik et al. (21) found that an increase in a company’s financial assets would reduce investment in R&D funding, thus affecting the development of the firm’s own main business.

Also, Li et al. (22) found that financialization has a squeeze-out effect on enterprise innovation output from the perspective of the nature of equity and market competition. Enterprises will be used for R&D and innovation funds for the financial sector, away from the main business, the pursuit of short-term interests, while ignoring the innovation process, resulting in a ‘crowding out effect ‘, leading to an independent innovation system is difficult to form, reduce innovation efficiency.

Pharmaceutical manufacturing innovation is a long-term process with high-risk and high-cost characteristics. Innovation efficiency can bring high profits for enterprises (23). Therefore, how to obtain innovation revenue has become the key. Innovation in China’s pharmaceutical manufacturing industry is inefficient (24). Part of the reason is that technological progress has not reached a certain level leading to insufficient innovation output. Another reason is that business managers invest money in the financial markets based on profit motives, making innovation resources scarce. Innovation efficiency is the input–output ratio of innovation activities, which needs the cooperation of manpower, material, and financial resources (25). Enterprises at any time extract innovative funds into the financial sector, resulting in impaired innovation sustainability, and increased innovation costs, thereby reducing innovation efficiency. In addition, the efficient innovation efficiency of enterprises is based on a certain scale of knowledge (26). Enterprises are addicted to financial profit-seeking behavior, which will lead to the decline of R&D personnel’s absorptive capacity, the decline of innovation knowledge accumulation, and the inhibition of innovation efficiency. Therefore, this paper puts forward the hypothesis:

*H1*: Corporate financialization has a negative impact on innovation efficiency.

### 2.3. The role of financing constraints in corporate financialization and innovation efficiency

When corporate financialization affects innovation, it may also be limited by the optimal financing level. When the degree of external financing constraints is high and the cost of financing is high, the motivation for corporate financialization is more preventive (27). Enterprises can seek more profits through the income of financial assets and play the role of reservoirs. Investing in financial products can increase internal retained earnings, ensure that enterprises have sufficient and continuous innovation capital investment, and guarantee innovation efficiency. Theurillat et al. (28) found that improving the allocation of financial assets across sectors and alleviating the financing difficulties of enterprises could promote business growth, which seems to indicate that when enterprises have financing constraints, corporate financialization does not inhibit the development of enterprises. Companies often use new investment channels to reduce financing costs, broaden financing channels and diversify business risks (29).

For enterprises, financing constraints essentially limit their innovation activities, more likely because of the lack of funds leading to the termination of innovation activities. When financing is complex, financialization is conducive to the accumulation of funds so that the innovation activities of enterprises have the continuity of capital injection. Tao et al. (30) investigated the social consequences of financialization, discovering that it has a squeezing effect on environmental investment. However, when funding is limited, financialization has a reservoir effect on ecological investment. The benefits of corporate financialization can ease financing constraints, reduce financing costs, and fund corporate innovation (31). By modeling the cash flow needs of firms, Almeida et al. (32) argued that corporate financialization can facilitate corporate innovation. When companies are profitable, financialization can ease financing difficulties and provide stable cash flow for companies ‘innovation activities. All of the preceding studies show that financing constraints will have an impact on the effect of corporate financialization on innovation. When a company faces financial constraints, the advantages of financialization can help them meet their needs for innovation funds, which will bring additional R&D funds to enterprises. It helps enterprises carry out the following innovation activities and provide financial resources to improve innovation efficiency. Based on this, this paper proposes the hypothesis:

*H2*: When enterprises have financing constraints, it will slow down the inhibitory effect of corporate financialization on innovation efficiency.

Some academics believe that the connection between financialization and innovation is not linear. Pan et al. (25) argued that financialization can significantly inhibit firm innovation, and the effect of financialization on innovation has a threshold. It implies that the degree of financialization can fluctuate within a reasonable range, which minimizes the “crowding out” effect of financialization on investments in innovation. Li et al. (33) had come to a similar conclusion. Wang et al. (34) found in the study that if the company’s “speculative” motivation is more potent, the effect of financialization crowding out enterprise innovation is more pronounced, but there is an inflection point between the two. This result suggests that the link between financialization and innovation will depend on the severity of funding restrictions. Guo (35) also started with the purpose of holding financial assets. When financing constraints are robust and corporate financialization has a limiting effect on investment in innovation. On the contrary, corporate financialization benefits investments in innovation. Hall (36) found that when large companies face insufficient R & D funds and high costs, unstable finance will limit businesses’ ability to innovate. At this time, venture financial investment becomes a solution to compensate for the funding gap. The funds obtained by enterprises through financial investment can make up for financing constraints, thus maintaining the innovation activities of enterprises. Enterprises’ decision-making on investments is impacted by financial constraints. When businesses experience severe financial restrictions, financial investment is more to keep the main business of enterprises, which can also ensure the stability of R&D funds and promote the innovation activities of enterprises. When enterprises face low financing constraints, financial investment is more profit-seeking motivation, which easily leads enterprises to ignore innovation activities, pursue short-term high returns, and reduce their innovation ability. Based on this, this paper proposes the hypothesis:

*H3*: The impact of corporate financialization on innovation efficiency has a threshold effect of financing constraints.

**Figure 1:**
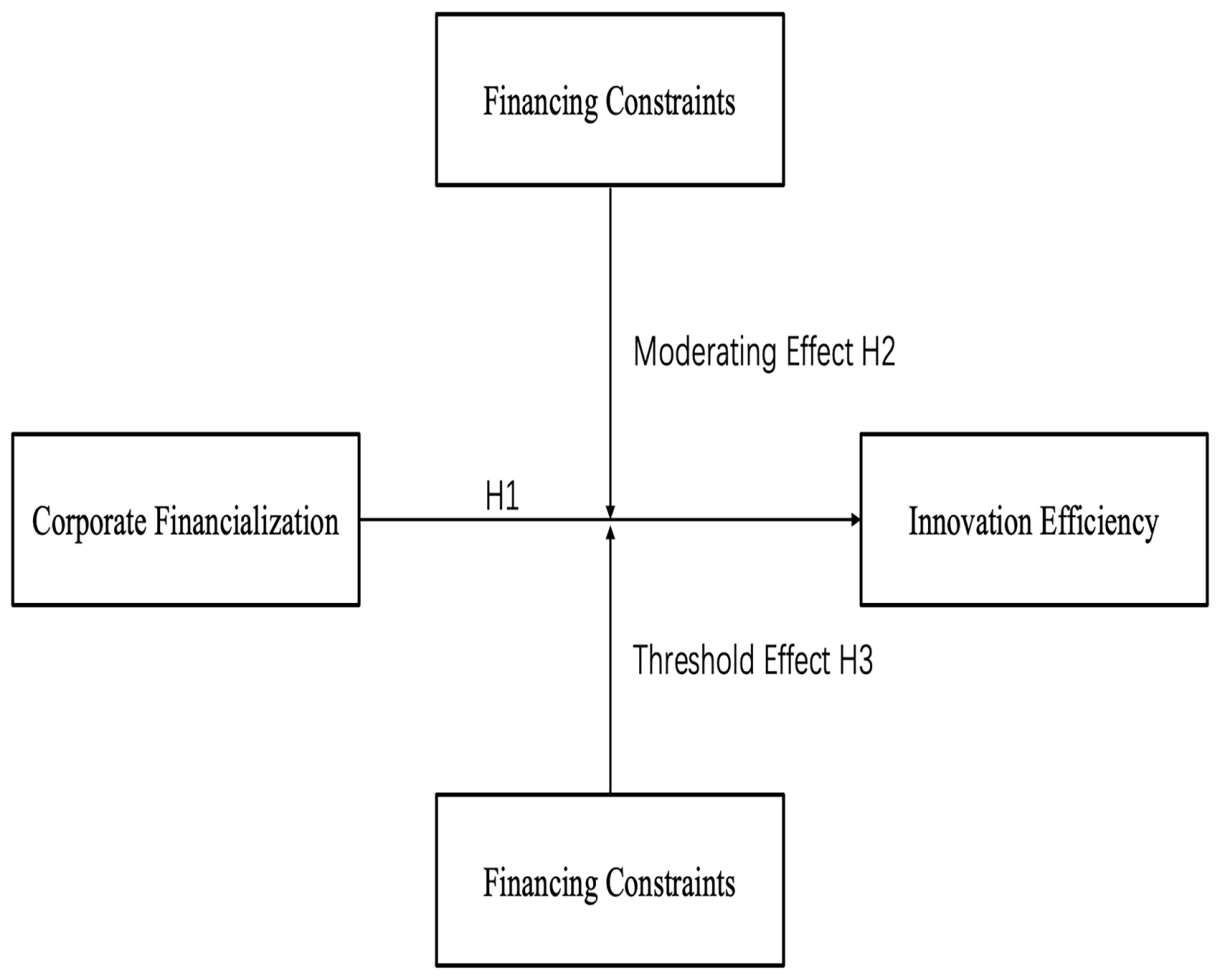
Research framework diagram.

Based on the above research hypotheses, this paper constructs a theoretical model of corporate financialization and innovation efficiency and also discusses the moderating and threshold effects of financing constraints. Figure 1 shows the research framework.

## 3. Materials and methods

### 3.1. Sample selection and data sources

The sample selected in this paper is China’s Shanghai and Shenzhen A-share pharmaceutical listed companies. Since the number of R&D personnel indicators was disclosed completely after 2015, the research period of this paper was selected as 2015–2020. This paper eliminates the data of listed companies with main missing variables, financial companies, and the samples of ST listed companies and constructs balanced panel data. The final sample includes 194 firms with a total of 1,164 observations. The data are mainly from the CSMAR database and CNRDS database. Data processing mostly used STATA 16.0 software.

### 3.2. Variables selection

#### 3.2.1. Dependent variable

There are three main ways of measuring innovation, namely innovation inputs, innovation outputs, and innovation efficiency. Innovation efficiency takes into account both innovation inputs and innovation outputs. Therefore, This study uses innovation efficiency (*TE*) to gauge how innovative a company is. The main methods and models for evaluating innovation efficiency are the use of non-parametric Data Envelopment Analysis (DEA) and parametric Stochastic Frontier Analysis (SFA). The principle of the efficiency calculation method is to construct the frontier based on econometric theory, and the deviation from the frontier is inefficient (37). Compared to non-parametric methods, Stochastic Frontier Analysis significantly considers the effects of environmental stochastic disturbances and management inefficiencies. SFA Aigner et al. (38) relied on the C-D production function. Existing studies have generally chosen R&D inputs and R&D personnel as indicators of innovation inputs and the number of patents as an indicator of innovation output (39, 40), The SFA model for calculating the innovation efficiency of firms in this paper is


$$Lnget_{it}=\alpha_{0}+\alpha_{1}Lnrd_{it}+\alpha_{2}Lnrdp_{it}+v_{it}-\mu_{it}$$

where *i* is an individual firm, *t* is time, $v_{it}-\mu_{it}$ denotes the random error term of the equation, $v_{it}$ is a random disturbance term and follows a normal distribution $N\left(0,\delta_{v}^{2}\right)$ , which represents the error of influence from uncontrollable human factors in the technical system, independent of $\mu_{it}$ . $\mu_{it}$ follows a truncated normal distribution $N\left(M_{it},\delta_{\mu }^{2}\right)$ , which enables the technical inefficiency values in the model to be measured. Get is the number of patents obtained. In this paper, we add 1 to the number of patents obtained and take the logarithm. *rd* is the ratio of R&D investment to operating revenue, and *rdp* is the number of R&D personnel. In this paper, we take the logarithm of both, respectively. The lower the value of technological inefficiency, the higher the number of patents obtained for a given amount of R&D investment and R&D staff.

Under the given technical level and factor input, technical efficiency can be defined as the ratio of real output to the maximum output represented by frontier, where frontier output is the output with technical efficiency loss $\mu_{it}$ equal to 0. Due to the influence of random noise and technical inefficiency, it is difficult for producers to reach the frontier level of production function in reality. Therefore, the technical efficiency embodied in the Stochastic Frontier Analysis of enterprise innovation efficiency $TE_{it}$ it is expressed as:


$$TE_{it}=\frac{E\left[f\left(x_{it},\beta \right)\exp\left(\upsilon_{it}-u_{it}\right)\right]}{E\left[f\left(x_{it},\beta \right)\exp\left(\upsilon_{it}-u_{it}\right)|u_{it}=0\right]}=\exp\left(-u_{it}\right)$$

where $f\left(x_{it},\beta \right)$ is the production frontier. Evidence that the parameter γ to be estimated can be used to test whether the model setting is reasonable is:


$$\gamma =\frac{\delta_{\mu }^{2}}{\delta_{\mu }^{2}+\delta_{v}^{2}}$$

According to the suggestion of Battese and Coelli (41), when 0≤γ≤1 , the SFA model is reasonable. The γ of this paper is 0.489, which meets the requirements.

**Figure 2:**
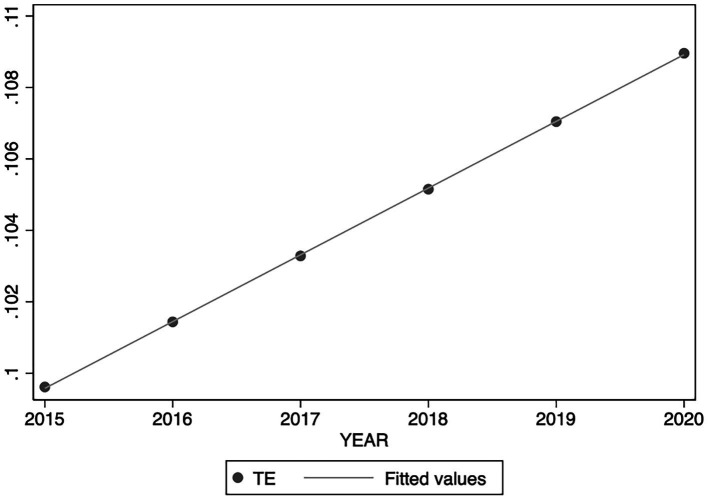
Average innovation efficiency of Chinese pharmaceutical-listed companies from 2015 to 2020.

Figure 2 shows the observed innovation efficiency value based on the above model. As can be seen, the mean value of innovation efficiency fluctuates between 0.05 and 0.15. The innovation efficiency value of Chinese pharmaceutical listed companies shows a steady upward trend. The efficiency value fluctuates between 0 and 1 (42). The maximum value of innovation efficiency is the production frontier value 1. At the same time, Lai et al. (6) used macro data to calculate the DEA value of innovation efficiency in the pharmaceutical industry. Its mean value is 0.289. The comparison between the two shows that the overall innovation efficiency of Chinese pharmaceutical listed companies is poor.

#### 3.2.2. Independent variable

This paper refers to (43). This paper includes financial assets held for trading, derivative financial assets, other current assets, net loans and advances granted, net available-for-sale financial assets, net held-to-maturity investments, and net investment properties as components of financial assets. This paper uses the proportion of financial assets in total assets to measure corporate financialization (*FIN*). At the same time, this paper selects the proportion of financial income (*RF*) to test the robustness, the enterprise’s ‘investment income ‘related to financial assets as a component of financial income and uses the proportion of financial income in total profits to horizontal financial income (*RF*).

#### 3.2.3. Moderating variable and threshold variable

There is an information asymmetry between the two sides of the supply of funds in the natural market environment, which limits enterprises’ access to external funding, thus creating a financing constraint. The essence of the above situation is the contradiction between the supply and demand of capital. The existing studies are more flexible in the way the financing constraint variables are taken. Representative measures are the KZ index, the WW index, and the SA index. To avoid the interference of endogeneity, Hadlock et al. (44) constructed an SA index following the KZ index approach, using two more exogenous variables, firm size(*size*) and firm age(*age*), which do not vary much over time. The SA index is calculated as $-0.737*lnsize+0.043*lnsize^{2}-0.04*age$ . Where *size* represents the natural logarithm of the enterprise’s total assets, and *age* represents the age of the enterprise.

#### 3.2.4. Control variables

Indicators of business financial features and corporate governance are chosen in this study as the control variables. The control variables are top ten shareholders’ shareholding (*TOP10*) (12), fixed assets ratio (*FAR*) (45), cash assets ratio (*CAR*) (45) and assets-liability ratio (*LEV*) (43). Where the top ten shareholders’ shareholding is measured as the sum of the shareholdings of the top ten shareholders, the fixed assets ratio is calculated using the percentage of net fixed assets to assets, the cash assets ratio is measured using the percentage of the balance of cash and cash equivalents to assets at the end of the period, and the assets-liability ratio is calculated using the ratio of liabilities to assets. The variables were selected and described as shown in Table 1.

**Table 1:** Variable selection and description.

Variable type	Variable	Symbols	Variable descriptions	Sources of variables
Dependent variable	Innovation efficiency	TE	Innovation efficiency of firms based on Stochastic Frontier Analysis production function measures	*get* is from CNRDS; *rd* and *rdp* from CSMAR
Independent variable	Corporate financialization	FIN	(Financial assets held for trading + derivative financial assets + other current assets + net loans and advances granted + net available-for-sale financial assets + net held-to-maturity investments + net investment properties) / total assets	CSMAR
	Proportion of financial income	RF	Financial income share/total profit	CSMAR
Moderating variable/threshold variable	Financing constraints	SA	$-0.737*lnsize+0.043*lnsize^{2}-0.04*age$	CSMAR
Control variables	Top ten shareholders’ shareholding	TOP10	Sum of the top ten shareholders’ shareholdings	CSMAR
	Fixed assets ratio	FAR	Fixed assets/total assets	CSMAR
	Cash assets ratio	CAR	Cash and cash equivalents balance at the end of the period /total assets	CSMAR
	Assets-liability ratio	LEV	Liabilities/assets	CSMAR

### 3.3. Model setting

#### 3.3.1. Benchmark regression model setting

To study the impact of corporate financialization on corporate innovation efficiency, the following model is constructed:


(1) $$\begin{array}{l} TE_{i,t}=\alpha_{0}+\alpha_{1}FIN_{i,t}+\alpha_{2}TOP10_{i,t}+\alpha_{3}FAR_{i,t}+\alpha_{4}CAR_{i,t}\\ \;\;\;\;\;\;\;\;\;\;\;\;\;\;\;\;\;+\alpha_{5}LEV_{i,t}+FirmFE+ProvinceFE+\varepsilon_{i,t} \end{array}$$

Among them, *i* represents the *ith* enterprise in the sample, *t* represents the year, *TE* represents the innovation efficiency of the enterprise, *FIN* represents the corporate financialization, *TOP10* represents the top ten shareholders’ shareholding, *FAR* represents the fixed asset ratio, *CAR* represents the cash asset ratio, *LEV* represents the asset-liability ratio, $\alpha_{0}$ represents the intercept term, *FirmFE* and *ProvinceFE* represent the fixed effects of the enterprise and the province respectively, $\alpha_{j}$ represents the coefficient, and $\varepsilon_{i,t}$ represents the error term.

#### 3.3.2. Moderating effect model setting

When enterprises face different financing constraints, corporate financialization will have different effects on innovation efficiency. To explore the different degrees of influence, this paper constructs the following moderating effect model. To avoid the impact of adjusting the cross-term on the multicollinearity of the regression results, this paper normalizes the cross-term.


(2) $$\begin{array}{l} TE_{i,t}=\beta_{0}+\beta_{1}FIN_{i,t}+\beta_{2}SA_{i,t}+\beta_{3}FIN_{i,t}*SA_{i,t}\\ \;\;\;\;\;\;\;\;\;\;\;\;\;\;\;\;+\beta_{4}TOP10_{i,t}+\beta_{5}FAR_{i,t}+\beta_{6}CAR_{i,t}+\beta_{7}LEV_{i,t}\\ \;\;\;\;\;\;\;\;\;\;\;\;\;\;\;\;+FirmFE+ProvinceFE+\varepsilon_{i,t} \end{array}$$

where $FIN_{i,t}*SA_{i,t}$ denotes the cross-multiplication term of corporate financialization and financing constraints, $\beta_{0}$ denotes the intercept term, $\beta_{j}$ denotes the coefficient, and the remaining symbolic meanings are the same as (1).

#### 3.3.3. Threshold effect model setting

Through the threshold model, under various levels of financial constraints, we can demonstrate how corporate financialization affects the effectiveness of innovation. To avoid errors in the subjective grouping, the threshold model (46) is used in this paper to validate.


(3) $$\begin{array}{l} TE_{i,t}=\gamma_{0}+\gamma_{1}FIN_{i,t}*I\left(SA_{i,t}\leq \theta \right)+\gamma_{2}FIN_{i,t}*I\left(SA_{i,t}>\theta \right)\\ \;\;\;\;\;\;\;\;\;\;\;\;\;\;\;\;\;+\gamma_{3}TOP10_{i,t}+\gamma_{4}FAR_{i,t}+\gamma_{5}CAR_{i,t}+\gamma_{6}LEV_{i,t}\\ \;\;\;\;\;\;\;\;\;\;\;\;\;\;\;\;\;+FirmFE+ProvinceFE+\varepsilon_{i,t} \end{array}$$

Among them, *TE* represents the dependent variable, *FIN* represents the crucial explanatory variable, *SA* represents the threshold variable, I(·) represents the indicator function, *θ* is the threshold value, $\gamma_{0}$ represents the intercept term, $\gamma_{j}$ represents the coefficient, and the rest of the symbols have the same meaning as Equation (1).

## 4. Empirical analysis results and discussion

### 4.1. Descriptive statistic

Descriptive statistics of the variables are shown in Table 2. It can be seen that the innovation efficiency (*TE*) of enterprises ranges from 0.009 to 0.690, with an average of 0.104, indicating that the innovation efficiency of enterprises is quite different, and the innovation efficiency of most pharmaceutical enterprises is low. The range of corporate financialization (*FIN*) is 0 ~ 0.620, and the average value is 0.079, indicating that corporate financialization (*FIN*) is mainly concentrated at about 10%, but the maximum value and the average value are quite different, meaning that corporate financialization (*FIN*) is quite different among enterprises. The value range of financing constraints (*SA*) is −4.502 ~ −3.307, the greater the value of SA, the greater the financing constraints, and the average value of financing constraints is −3.889. It can be seen that enterprises generally face the plight of financing constraints.

**Table 2:** Descriptive statistics.

Variables	*N*	Mean	SD	Min	Max
TE	1,164	0.104	0.101	0.009	0.690
FIN	1,164	0.079	0.095	0	0.620
RF	1,164	0.037	0.357	−1.273	7.743
SA	1,164	−3.889	0.200	−4.502	−3.307
TOP10	1,164	56.920	13.855	19.830	100
FAR	1,164	0.197	0.109	0.002	0.663
CAR	1,164	0.165	0.111	0.001	0.676
LEV	1,164	0.329	0.177	0.014	0.990

### 4.2. Relevance analysis

The results of the Pearson correlation analysis between the variables are presented in Table 3. The results show that there is a significant negative correlation between innovation efficiency and corporate financialization, and a significant positive correlation between financial income and corporate financialization, indicating that with the increase of financial income, the proportion of corporate financialization will also increase, and enterprises will increasingly invest in the financial sector.The findings demonstrate that the variance inflation factor VI*F* values are all less than 10, whereas the Pearson correlation coefficients for the primary variables are all less than 0.8. It demonstrates that multicollinearity between variables is not a concern.

**Table 3:** Relevance analysis.

	TE	FIN	SA	RF	TOP10	FAR	CAR	LEV	VIF
TE	1.000								
FIN	−0.049*	1.000							1.15
SA	−0.081***	0.048*	1.000						1.06
RF	0.007	0.107***	−0.077***	1.000					1.01
TOP10	0.033	0.040	0.201***	−0.113***	1.000				1.07
FAR	−0.044	−0.160***	0.024	0.019	−0.080***	1.000			1.11
CAR	−0.096***	−0.040	0.020	−0.027	0.147***	−0.241***	1.000		1.22
LEV	0.158***	−0.274***	−0.138***	0.041	−0.110***	0.019	−0.293***	1.000	1.24

Robust *t*-statistics in parentheses, ***p < 0.01, **p < 0.05, *p < 0.1.

### 4.3. Baseline regression analysis

Panel data can avoid unobservable individual effects. This paper uses balanced panel data for empirical analysis. By performing the F test, BP test, and Hausman test on sample data, the fixed effect model is finally selected. Existing research literature believes Peng et al. (47) that corporate financialization has a lag effect. To examine more comprehensively, this paper also substitutes the lagged term of corporate financialization into the model for regression and uses the fixed effect model to benchmark the impact of corporate financialization on innovation efficiency. The results are shown in Table 4.

**Table 4:** Benchmark regression results and robustness test.

Variables	(1)	(2)	(3)	(4)
	TE	TE	Tobit regression	Robustness test
FIN	−0.100*** (−5.36)		−0.141** (−2.12)	
L.FIN		−0.057*** (−3.36)		
RF				−0.011*** (−3.39)
TOP10	−0.002*** (−8.69)	−0.002*** (−8.71)	−0.003*** (−6.36)	−0.002*** (−9.14)
FAR	−0.085*** (−3.81)	−0.022 (−0.93)	−0.208*** (−3.32)	−0.064*** (−2.85)
CAR	−0.053*** (−3.48)	−0.009 (−0.54)	−0.108^***^ (−2.89)	−0.034^**^ (−2.25)
LEV	−0.002 (−0.15)	0.001 (0.12)	−0.011 (−0.32)	−0.004 (−0.31)
Constant	0.137*** (9.75)	0.126*** (8.79)	1.506 (0.02)	0.131*** (9.25)
Observations	1,164	970	1,164	1,164
Provincial Fixed Effect	Yes	Yes	Yes	Yes
Enterprise Fixed Effect	Yes	Yes	Yes	Yes
R-squared	0.121	0.113	0.457	0.105

Robust *t*-statistics in parentheses, ***p < 0.01, **p < 0.05, *p < 0.1.

The coefficient of corporate financialization (*FIN*), which can be observed in Column (1), is 0.102 and reaches the significance level of 1%. Corporate financialization has a detrimental effect on the effectiveness of innovation, which verifies Hypothesis 1. On this basis, the financialization of enterprises lags for one period. Column (2) shows that the financialization coefficient for businesses that are one period behind (*L.FIN*) is 0.058, which satisfies the significance level of 1%. The financialization of enterprises lagging one period still negatively affects the innovation efficiency, and the influence coefficient is declining, indicating that corporate financialization has a specific time lag and persistence. This shows that through a period of corporate financial investment, the negative effects of corporate financialization are lessening, and there is a specific accumulation of surplus capital within the company to support financial R&D of innovation activities. Although the negative effect is falling, enterprises invest resources in the financial market, which will make funds withdrawn from the operating assets of enterprises. The R&D investment of firms is relatively reduced, the resulting patent output will also decrease, and the impact on innovation efficiency is still negative. This demonstrates the incentive of profit-seeking. Businesses invest money in the financial market in hopes of earning quick returns, squeezing out the funds and necessary resources needed for enterprise innovation. The profits brought by financial investment do not bring significant feedback to the technological innovation of enterprises, but affect the efficiency of capital allocation and the efficiency of innovation, which is detrimental to the industry as a whole.

### 4.4. Robustness test

The innovation efficiency is a continuous percentage variable between 0 and 1, and there should be ‘0 value ‘or ‘1 value ‘. However, the innovation efficiency of this paper is between 0.009 and 0.690, which is a truncated random variable. Since the variable is discretely distributed, it is a restricted explained variable. Using only panel fixed effects model estimates may produce biased and inconsistent results (48). To maintain the robustness of estimation, this paper uses the panel Tobit model to estimate. The results are shown in Table 4.

It can be seen from Column 3 that the coefficient of corporate financialization (*FIN*) is −0.141, which is significant at the 5% level. Corporate financialization negatively affects innovation efficiency. It is reliable and agrees with the benchmark regression results. For the measurement of financialization, this paper uses the financial return ratio (*RF*) to replace the explanatory variables for robustness analysis. As shown in column 4, the coefficient of financial return ratio (RF) is-0.072, which meets the significance level of 1%, which is consistent with the benchmark regression results and is robust.

### 4.5. Endogeneity test

Corporate financialization will impede the innovative efficiency of firms, but enterprises with high innovation efficiency will have a lot of rich funds to make the financial investment and expect short-term returns. This suggests that business financialization and innovation effectiveness may be related in a two-way causal manner. To prevent any endogeneity issues, this work introduces the instrumental variable approach using the two-stage regression method (2SLS) for the measurement test. According to (49), this paper uses the logarithm of cash paid for the purchase and construction of fixed assets, intangible assets, and other long-term assets(*INVEST*) as an instrumental variable. First of all, the higher investment spending on fixed assets, intangible assets, and other long-term assets of enterprises, the fewer funds enterprises invest in virtual financial markets, so the variable meets the correlation requirements. For the exclusive constraint of instrumental variables, that is, exogeneity, no recognized article can statistically test the exogeneity of instrumental variables. This article refers to Acemoglu et al. (50). Since there is only one instrumental variable in this paper, an over-identification test is not required. Therefore, the exogeneity of instrumental variables is demonstrated from two aspects: Firstly, according to the ‘*Chinese Enterprise Accounting Standards*’, corporate investment is divided into inward investment and outward investment according to different investment directions. Inward investment is the fixed assets, intangible assets and other long-term assets needed by enterprises to provide funds for their main business such as production and operation. Outward investment is the purchase of securities or other financial products by enterprises for financial investment. Therefore, the instrumental variable selected in this paper is the direction of internal investment of enterprises, and the two do not coincide. Secondly, this paper adds the control variables of the company’s business decision-making related to the explanatory variables to demonstrate the robustness of the results. Relevant control variables are current ratio (CR), quick ratio (QR), net profit growth rate (PGR), current asset ratio (CCAR) and proportion of independent directors (ID). It can be seen from Table 5 that expected regressions in columns 3 and 4 have not changed significantly. Therefore, this paper can still use this instrumental variable. Finally, this paper tests the validity of instrumental variables. The null hypothesis that there are no soft instrumental factors is rejected when the F statistic in the weak instrumental variable test is larger than 10. In the heteroscedasticity DWH test results, the *value of p* is less than 0.01, indicating that corporate financial investment behavior is an endogenous explanatory variable. The model excludes the possibility of endogeneity.

**Table 5:** 2SLS regression results.

Variables	(1)	(2)	(3)	(4)
	IV-II	IV-I	IV-II	IV-I
FIN	−0.580*** (−4.21)		−0.574*** (−4.22)	
INVEST		−0.018*** (−4.68)		−0.018*** (−4.73)
TOP10	−0.002*** (−4.59)	0.001* (1.83)	−0.002*** (−4.61)	0.001** (2.00)
FAR	−0.181*** (−4.53)	−0.241*** (−5.28)	−0.189*** (−4.63)	−0.252*** (−5.49)
CAR	−0.140*** (−4.20)	−0.206*** (−5.92)	−0.140*** (−4.17)	−0.210*** (−6.02)
LEV	0.002 (0.15)	0.007 (0.29)	0.002 (0.14)	0.008 (0.32)
CR			−0.004 (−1.63)	−0.007** (−1.90)
QR			0.003 (0.95)	0.005 (1.05)
PGR			0.000 (0.49)	0.000 (0.56)
CCAR			0.009 (0.74)	0.023 (1.33)
ID			−0.007* (−1.77)	−0.004 (−0.76)
Constant	1.502*** (46.54)	0.392*** (4.60)	1.527*** (40.11)	0.340*** (4.67)
*R*-squared	0.998	0.674	0.998	0.678
*F*	22.511***		9.611***	
*P*	0.000		0.001	
Provincial fixed effect	Yes		Yes	
Enterprise fixed effect	Yes		Yes	

Robust *t*-statistics in parentheses, ***p < 0.01, **p < 0.05, *p < 0.1.

Table 5 reports the regression results of the 2SLS estimation method for instrumental variables. In the first stage, it also has been discovered that firms’ investment practices significantly reduce the effectiveness of innovation (the coefficient is −0.018, *p* < 0.01) in column 1. At the same time, the regression results and benchmark results of corporate financialization remain stable. The aforementioned findings confirm once more that corporate financial investment practices have a deterrent influence on innovation effectiveness.

### 4.6. Heterogeneity analysis

#### 4.6.1. Sub-regional testing

The uneven development between regions in China and regional factors may also affect the innovation efficiency of firms. This paper classifies the sample companies according to the location of their offices obtained from the CSMAR database into Eastern, Central and Western companies according to the NBS classification. Finally, 131 companies from the East, 30 companies from the Central and 33 companies from the West were included in the sample.

Table 6 displays the effects of the company financialization regression on innovation efficiency in the eastern, central, and western regions. The financialization of enterprises in the western region has the most significant negative impact on innovation efficiency, followed by the central and eastern regions, as can be seen from the correlation between corporate financialization and innovation efficiency of enterprises in the eastern, central, and western regions. This shows that compared with the central and east regions, the development of enterprises in the western region is relatively weak. Less talent and lack of funds lead to financial investment behavior to a greater extent occupying the enterprise’s innovation funds. Enterprises seek short-term interests and ignore the development of innovation activities, which is not conducive to improving innovation ability and leads to declining innovation efficiency. The eastern and central regions’ finance practices do not support businesses’ ability to innovate effectively, but due to the relatively high degree of financial development, the degree of financing convenience is higher. The types of financial assets available are broader, and the capital market is more perfect. Therefore, financialization has a negligibly small impact on company innovation investment, a negligibly small impact on innovation output, and a negligibly small impact on innovation efficiency.

**Table 6:** Sub-regional regression results and regression results of enterprise nature.

Variables	(1)	(2)	(3)	(4)	(5)
	TE	TE	TE	TE	TE
	Eastern region	Central region	Western region	State-owned enterprises	Non-state-owned enterprises
FIN	−0.074*** (−3.29)	−0.097* (−1.89)	−0.203*** (−4.51)	−0.209*** (−3.15)	−0.089*** (−4.60)
TOP10	−0.002*** (−6.54)	−0.003*** (−6.70)	−0.001** (−2.09)	0.000 (0.30)	−0.002*** (−9.43)
FAR	−0.091*** (−3.16)	−0.155** (−2.61)	−0.066 (−1.43)	−0.129** (−2.29)	−0.076*** (−3.09)
CAR	−0.056*** (−2.99)	−0.021 (−0.47)	−0.061* (−1.84)	−0.046 (−1.06)	−0.056*** (−3.42)
LEV	−0.004 (−0.27)	0.068* (1.92)	−0.023 (−0.77)	0.031 (0.77)	−0.007 (−0.48)
Constant	0.086*** (4.96)	0.302*** (9.35)	0.171*** (4.79)	0.165*** (3.23)	0.114*** (7.94)
Observations	786	180	198	222	942
Provincial fixed effect	Yes	Yes	Yes	Yes	Yes
Enterprise fixed effect	Yes	Yes	Yes	Yes	Yes
*R*-squared	0.092	0.276	0.204	0.064	0.152

Robust *t*-statistics in parentheses, ***p < 0.01, **p < 0.05, *p < 0.1.

#### 4.6.2. Sub-business nature test

To explore the differences in the corporate financialization on innovation among enterprises with different property rights, the sample businesses were split into state-owned and privately owned businesses to assess heterogeneity. Table 6 displays the results of the regression.

Both state-owned and non-state-owned firms’ financialization has a large negative impact on innovation efficiency, with state-owned enterprises’ negative impact on innovation efficiency being greater than that of non-state-owned enterprises. This could be because state-owned businesses typically have more serious agency issues than non-state businesses, more accessible access to credit from external financial institutions, and can alleviate the problem of corporate information asymmetry through regulatory channels and information channels. Therefore, based on the advantages of information and enterprise nature, managers of state-owned companies are more eager to make investments in the financial markets. However, managers of state-owned enterprises often lack ownership incentives and have more vital term performance considerations. Innovation investment is intertemporal and uncertain. The large amount of capital invested by managers during their tenure often cannot ensure stable returns. Therefore, the management of state-owned enterprises does not have enough enthusiasm to engage in innovation activities. They often increase financial market investment, obtain short-term returns, and reduce or even abandon investment in R&D and innovation because of short-term performance pressure, which leads to a lack of innovation results and low innovation efficiency. For non-state-owned enterprises, enterprises need continuous innovation to maintain their market share. Therefore, their willingness to innovate is strong. The income obtained by enterprises investing in financial markets has a higher conversion rate to innovation efficiency, and the negative effect of financialization on innovation efficiency is lower.

### 4.7. Further testing

#### 4.7.1. Test on the moderating effect of financing constraints

Considering the study that has already been done on the connection between corporate financialization and innovation, the theoretical analysis of this paper believes that financing constraints are used as a moderating variable for corporate financialization to affect innovation efficiency. Under this assumption, this paper argues that listed companies in China’s pharmaceutical industry are subject to financing constraints. Based on this, this paper uses two methods to verify the existence of financing constraints in Chinese pharmaceutical listed companies, namely direct method and indirect method. The indirect method is to verify the cash-cash flow sensitivity. This paper refers to Almeida et al. (32) practice and constructs the following benchmark model:


(4) $$\Delta CashHoldings_{i,t}=\delta_{0}+\delta_{1}CashFlow_{i,t}+\delta_{2}Q_{i,t}+\delta_{3}Size_{i,t}+\varepsilon_{i,t}$$

Among them, *ΔCashHoldings* represents the change of the annual cash holdings of the enterprise, *CashFlow* represents the cash flow of the enterprise, *Q* represents the investment opportunity of the enterprise, *Tobin’s Q* represents *Q*, *Size* represents the size of the company, $\delta_{0}$ represents the intercept term, $\delta_{j}$ represents the coefficient, and the remaining symbols mean the same as (1). where CashHoldings = cash and cash equivalents/total assets; cashFlow = net operating cash flow/total assets; Q = total market value/total assets; SIZE = the natural logarithm of total assets. The data are from CSMAR and processed by tail reduction.

According to the above model, if the company faces financing constraints, $\delta_{1}$ (a measure of cash-cash flow sensitivity coefficient) is significantly positive; non-financing constraint companies did not show significance. To test the above hypothesis, we need a standard to distinguish whether the company is subject to financing constraints. This paper refers to Lian et al. (51) practice and selects the following two standards as constraint variables:

(1) Payout ratio. In this paper, the data is divided into three tiers according to the payout ratio in three quartiles. The companies in the highest quantile are considered to be non-financing constrained companies, and the companies in the lowest quantile are financing constrained companies.

(2) Company size. In this paper, the data are divided into three levels according to the company size. The company at the highest quantile is considered to be a financing constraint company, and the company at the lowest quantile is a non-financing constraint company.

The results of the benchmark regressions are given in Table 7. The results find that the *CashFlow* coefficient is significantly positive, which is in line with theoretical expectations, whether payout rate or company size is used as the basis for grouping. In contrast, the sensitivity to *Cashflow* without financing constraints is not significant. This indicates that the use of the direct method can demonstrate the existence of financing constraints for listed Chinese pharmaceutical companies.

**Table 7:** Proof of the existence of financing constraints benchmark regression results.

Dependent variable	Independent variables			
ΔCashHoldings	CashFlow	*Q*	Size	$R^{2}$
Financial constraints criteria				
1. Payout ratio				
Constrained firms	0.166* (1.76)	−0.031*** (−3.23)	−0.023* (−1.92)	0.131
Unconstrained firms	0.190 (1.61)	−0.004 (−1.36)	−0.010 (−0.56)	0.013
2. Firm size				
Constrained firms	0.211** (2.38)	−0.002 (−0.68)	0.161 (1.15)	0.014
Unconstrained firms	−0.050 (0.740)	−0.006* (−1.71)	−0.128*** (−3.58)	0.012

Robust *t*-statistics in parentheses, ***p < 0.01, **p < 0.05, *p < 0.1.

The direct method is to compare the financing constraints of listed companies in China’s pharmaceutical industry and other industries. This paper refers to the 2021 standard of Shenyin Wanguo Industry Classification^1^, and compares the financing constraints of the pharmaceutical industry and other industries by mean T test. The KZ index is calculated by Kaplan et al. (52), and the WW index is calculated by Whited et al. (53). Among them, the SA index, KZ index and WW index, the greater the value, the greater the financing constraints. It can be seen from Table 8 that the average value of SA index and WW index in the pharmaceutical industry is significantly higher than that in other industries, indicating that listed companies in the pharmaceutical industry have financing constraints.

**Table 8:** Comparison of financing constraint values.

	Pharmaceutical industry averages	Other industry averages	*t* values	*p* values
SA	−3.889	−4.021	13.685	0.000
KZ	0.097	1.649	−16.016	0.000
WW	−0.827	−1.061	16.665	0.000

Based on the indirect method and the direct method, this paper comprehensively believes that there are financing constraints in Chinese pharmaceutical listed companies. This verifies the moderating effect of financing constraints.

This part introduces the intersection of corporate financialization and financing constraints. It empirically examines whether financial limitations can lessen the negative impact that corporate financialization has on the effectiveness of innovation. Table 9 presents the outcomes. It can be seen from Column (2) that the coefficient of corporate financialization (*FIN*) is −0.111, which meets the significant level of 1%. The coefficient of the cross term (FIN* SA) is 0.155, and it meets the 10% threshold for significance. This demonstrates how financial limitations moderate the relationship between corporate financialization and innovation efficiency. The detrimental impact of corporate financialization on innovation efficiency is lessened as a result of tighter financial restrictions, which verifies Hypothesis 2. At the same time, this paper uses the KZ index and the WW index to verify the moderating effect of financing constraints again, as shown in Table 9 columns (3) and columns (4).

**Table 9:** Further test.

Variables	(1)	(2)	(3)	(4)
	TE	TE	TE	TE
	Threshold regression	Moderating effect	Robustness test	Robustness test
FIN(SA ≤ −4.029)	−0.213*** (−6.29)			
FIN(SA > −4.029)	−0.081*** (−4.18)			
FIN		−0.110*** (−5.70)	−0.107*** (−5.72)	−0.057** (−2.04)
SA/KZ/WW		0.002 (0.13)	0.002** (2.34)	0.019*** (4.33)
FIN*SA/FIN*KZ/FIN*WW		0.166** (2.02)	−0.022*** (−3.32)	0.054** (1.93)
TOP10	−0.002*** (−8.98)	−0.002*** (−8.71)	−0.002*** (−8.24)	−0.002*** (−8.54)
FAR	−0.088*** (−3.95)	−0.088*** (−3.92)	−0.085*** (−3.84)	−0.082*** (−3.78)
CAR	−0.057*** (−3.76)	−0.053*** (−3.46)	−0.040** (−2.35)	−0.047*** (−3.14)
LEV	−0.002 (−0.17)	−0.002 (−0.14)	−0.002 (−0.18)	−0.002 (−0.17)
Constant	0.139*** (10.10)	0.145*** (2.66)	0.129*** (9.08)	0.146*** (10.46)
Observations	1,164	1,164	1,164	1,164
Provincial fixed effect	Yes	Yes	Yes	Yes
Enterprise fixed effect	Yes	Yes	Yes	Yes
*R*-squared	0.129	0.118	0.131	0.173
Empirical *p* value	0.000***			

Robust *t*-statistics in parentheses, ***p < 0.01, **p < 0.05, *p < 0.1. The “Empirical p value” was used to test the significance of the difference in Fisher Combination Test coefficient between groups, which was obtained by Bootstrap 1,000 times. The variable line4 SA/KZ/WW corresponds to the column (2) moderating variable SA index, the column (3) moderating variable KZ index, and the column (4) moderating variable WW index. Similarly, the variable line5 FIN *SA/FIN*KZ/FIN*WW.

It can be inferred that the financing constraints enterprises faced largely determine the impact of corporate financialization on innovation efficiency. The influence of corporate financialization on innovation efficiency is modest if businesses suffer substantial funding restrictions. The reason is that when the enterprise is seriously short of funds, a small part of the funds will be invested in the financial market to obtain short-term benefits. It is conducive to supplementing funds for enterprise innovation activities and avoiding the suspension of business activities due to serious financing difficulties. If faced with loose financing constraints, the inhibitory effect is more substantial. At this time, enterprises invest in financial markets for profit-driven motives, with financial investment to solve the burden of enterprises more to obtain financial returns. Managers’ short-sighted behavior often makes financialization crowd out the innovation investment of enterprises, thereby reducing the output of innovation results and inhibiting innovation efficiency.

**Figure 3:**
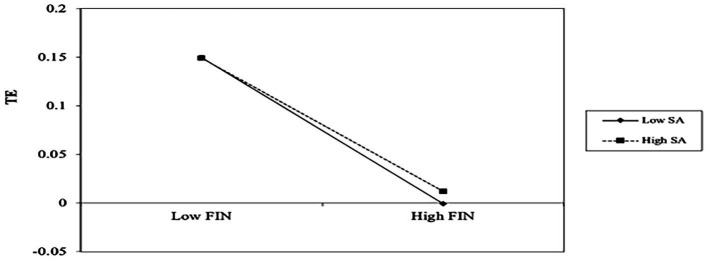
Moderating effect of financing constraints.

To further explain the above empirical results, this paper draws the adjustment curve, as shown in Figure 3. The detrimental impact of business financialization on innovation efficiency is lessening as the level of financial restraints increases.

#### 4.7.2. Threshold effect test of financing constraints

From the above adjustment affect results, it is clear that financial constraints can lessen corporate financialization’s negative influence on innovation efficiency, demonstrating the fact that corporate financialization’s effects on innovation efficiency depend on the amount of financing available. Therefore, this study puts the non-linear link between business financialization and innovation effectiveness to the test using the threshold effect.

In this paper, the asymptotic value of the F-statistic is obtained using the Bootstrap test for threshold effects, the value of p is obtained, and the threshold is tested at 400 grid searches and 300 bootstrap samples, respectively. The results are shown in Table 10. This paper refers to Wang et al. (13) practice. When the threshold variable is the company financialization itself, the company financialization has a significant single threshold effect on the innovation efficiency, which shows that the company financialization and innovation efficiency have a non-linear relationship. Then this paper verifies that corporate financialization has a significant single threshold effect on innovation efficiency when the threshold variable is financing constraints, with a threshold value of-4.029. The F statistic of the single threshold is 18.37, which is effective at the 1% significance level. The double threshold and the triple threshold have not passed the test.

**Table 10:** Threshold effect test results.

Independent variable	Threshold variable	Hypothesis testing	RSS	MSE	*F* value	*p* value	Threshold value	95% Confidence interval
FIN	FIN	Single threshold	0.7433	0.006	18.78	0.033	0.003	[0.0027, 0.0030]
FIN	FIN	Double threshold	0.7386	0.001	7.39	0.530	0.005	[0.0045, 0.0047]
FIN	FIN	Triple threshold	0.7339	0.001	7.37	0.833	0.015	[0.0153, 0.0154]
FIN	SA	Single threshold	1.181	0.001	18.37	0.000	−4.029	[−4.053, −4.027]
FIN	SA	Double threshold	1.175	0.001	5.46	0.3233	−4.069	[−4.107, −4.068]
FIN	SA	Triple threshold	1.171	0.001	4.24	0.5900	−4.103	[−4.104, −4.103]

**Figure 4:**
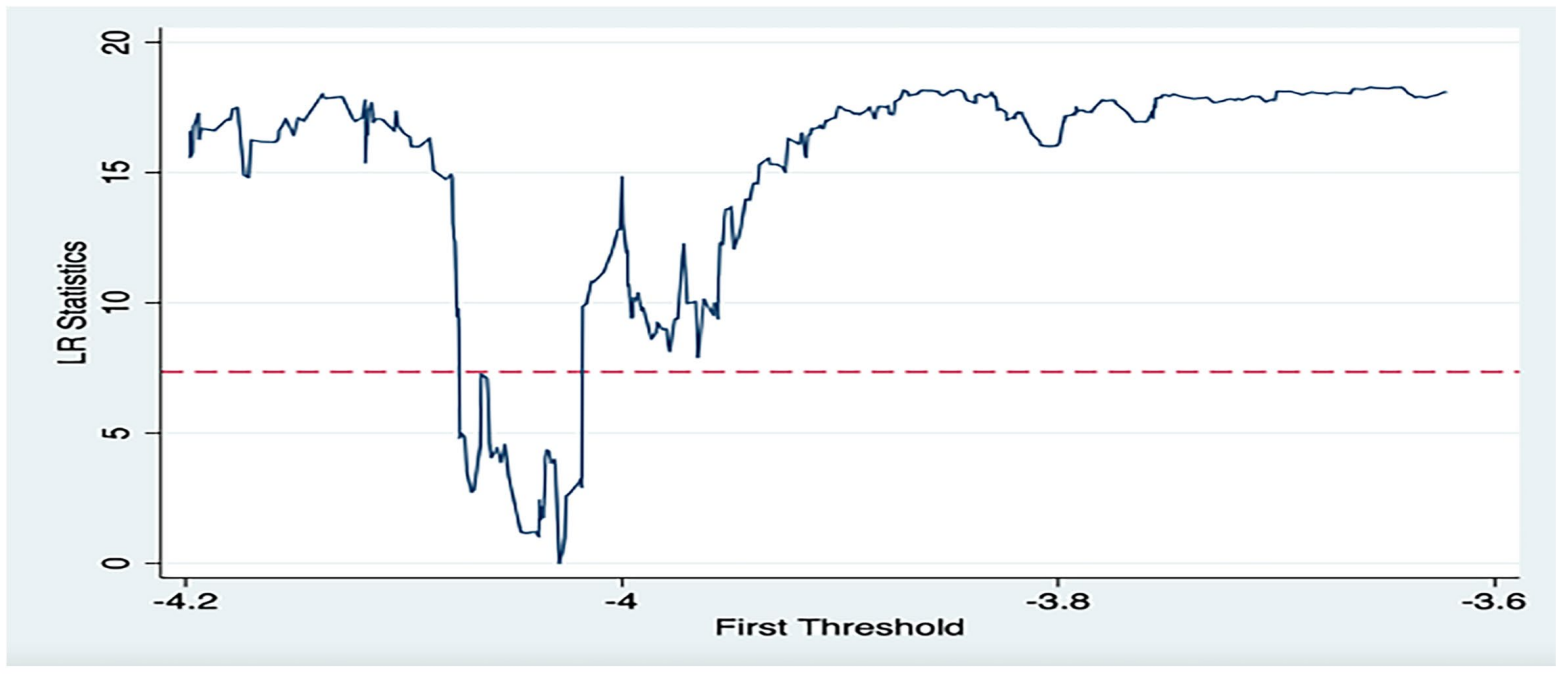
Likelihood ratio function of threshold effect test.

The likelihood ratio function was used to confirm the accuracy of the threshold estimations and to provide a more intuitive understanding of the threshold effect and 95% confidence interval construction. The outcomes are displayed in Figure 4. The likelihood ratio statistic LR value is zero when the threshold is assessed to be −4.029 for all LR values, the threshold estimate’s 95% confidence interval is less than 5% for all LR values. The gap formed by the threshold at the significance level indicates that the threshold estimate equals the actual value.

Further parameter estimation of the single threshold model can obtain the threshold regression results of financialization and innovation efficiency. Table 9 presents the outcomes. It can be seen from Column (1) that the finance constraint (*SA*) is smaller than −4.029, as can be demonstrated. Corporate financialization has a negative impact on innovation efficiency, the coefficient is −0.213, significant at the 1% level; when the financing constraint (*SA*) is greater than −4.029, corporate financialization also has a negative impact on innovation efficiency, the coefficient is −0.081, significant at the 1% level. This shows a non-linear negative correlation between corporate financialization and corporate innovation efficiency. With the increase of financing constraints, the negative impact is weakened, which verifies Hypothesis 3. In order to verify the significance of the difference between the coefficient groups, this paper refers to Lian et al. (54) practice, and uses the Fisher Combination Test to verify the difference of the coefficient. Through 1,000 self-sampling, the empirical *p* value is 0.000, indicating that the coefficient is different. It can be seen that when the financing constraints of enterprises jump over a certain threshold, financialization’s inhibitory effect on innovation efficiency decreases, which is also in line with the results of the above moderating effect. When the financing constraints of enterprises are small, at this time, enterprises are more likely to replace long-term innovation investment with financial speculation for the purpose of short-term arbitrage, and gain income through rapid cash return. This profit-seeking behavior makes enterprises lose the motivation of innovation investment, damage the enthusiasm of R&D personnel, and have a greater inhibitory effect on innovation efficiency. When the financing constraints of enterprises are large, the surplus funds of enterprises are less, and enterprises will not place funds on higher risk financial investment when they face operational difficulties. In order to convey a good business strategy signal to the outside world, enterprises invest funds in innovation activities. Even if at this time, the financial investment of enterprises is more to maintain the innovation activities of enterprises, not based on the short-term performance of enterprises, so the inhibition of innovation efficiency is smaller.

## 5. Conclusion and discussion

Currently, Chinese enterprises are fully implementing innovation-driven, and the innovation ability of pharmaceutical enterprises is constantly improving. As a representative entity industry, the pharmaceutical manufacturing industry has become an essential factor affecting national and regional development. When pharmaceutical enterprises face financing difficulties, the impact of corporate financialization on innovation efficiency is a critical issue in innovation-driven development. Based on the sample data of Chinese pharmaceutical listed companies from 2015 to 2020, this paper empirically analyzes the impact of corporate financialization on innovation efficiency and the moderating effect and threshold effect of financing constraints in the relationship between them. The final conclusions are as follows:① Overall, there is a negative impact of corporate financialization in the current and lagging periods on innovation efficiency. That is, financial investment inhibits the innovation efficiency of firms.② Based on the grouping test of different regions and enterprise nature, corporate financialization’s impact on innovation efficiency is significantly harmful. The inhibitory effect of corporate financialization on innovation efficiency is more evident in the western region than in the eastern and central regions and more evident in state-owned enterprises than non-state-owned enterprises. ③After further mechanism tests, it is found that financing constraints show a moderating effect in the process of corporate financialization affecting innovation efficiency. That is, the impact of corporate financialization on innovation efficiency decreases with the increase of financing constraints. ④The impact of corporate financialization on innovation efficiency has a threshold effect of financing constraints. When the level of financing constraints crosses the threshold value, the negative impact of corporate financialization on innovation efficiency is weakened.

## 6. Policy proposal

Corporate financialization can bring short-term benefits to enterprises, but it will also have a negative impact on innovation efficiency. Based on the above conclusions, this paper puts forward the following four suggestions: First, corporate financialization will inhibit the innovation efficiency of enterprises. Therefore, enterprises should determine the appropriate level of financialization, coordinate the relationship between financial asset holding and innovative business in operation, reasonably set the proportion of R&D assets and financial assets in asset allocation, clarify the strategic positioning of enterprises, and avoid the loss of innovation ability caused by excessive financialization. Enterprises should strictly control the share of financial assets, encourage enterprises to use disposable funds for R&D innovation that can bring long-term development, and provide original funds to improve innovation efficiency. Second, compared with the eastern and central regions and non-state-owned enterprises, the corporate financialization of the western region and state-owned enterprises has a greater negative impact on innovation efficiency. Enterprises in the west and central regions should improve their innovation awareness and create a good atmosphere for innovation. Enterprises should invest capital accumulation in innovation activities rather than financializing funds to squeeze R&D investment for speculative purposes. In corporate governance, companies should strengthen the supervision of managers to reduce the possibility of short-sighted investment behavior. Enterprises can set up a reasonable long-term incentive mechanism to guide managers to allocate resources to projects such as technological innovation that can promote the improvement of enterprise value. For example, enterprises regard innovation performance as an essential part of performance appraisal to encourage innovation efficiency and achieve sustainable development of enterprises. Third, business funding restrictions are one of the primary factors limiting corporate innovation. Since financing constraints can alleviate the negative impact of corporate financialization on innovation efficiency, enterprises should allocate funds appropriately. Internal financing is the most convenient way of financing, which can often alleviate financing constraints. Therefore, enterprises should accumulate funds in advance for their innovation activities. The rational use of idle funds by enterprises can effectively improve the innovation efficiency of enterprises, achieve the balance between corporate financialization and innovation efficiency, and provide sufficient funds for the R&D of enterprises. Fourth, enterprises need to have a sound risk prevention mechanism system. Under different levels of financing constraints, the impact of corporate financialization on innovation efficiency is different. When financing constraints are more incredible, the inhibitory effect of corporate financialization on innovation efficiency is reduced. At this time, the enterprise’s financial investment can be used as a risk response measure. When the financing gap of enterprises is not large, the financial investment of enterprises at this time can not alleviate the shortage of R&D funds. Therefore, when facing different financing difficulties, enterprises can make up for innovation funds through different risk response strategies, invest carefully, and achieve effective incentives for innovation efficiency.

## 7. Limitations and future work

This study also has limitations. First, corporate financialization is primarily related to managers’ decision-making. This paper does not include managers’ preferences in the research object and managers’ preferences can also be included in future research; Second, although the pharmaceutical manufacturing industry is a typical representative of the real economy and high-tech economy, the research object is still limited. Future research can take into account all manufacturing industries; Third, due to the availability of data, this paper only considers listed pharmaceutical companies, but non-listed pharmaceutical manufacturing companies also have financing difficulties and financialization. Future research can turn to non-listed companies in order to fully understand the relationship between financialization and innovation.

## Data availability statement

Publicly available datasets were analyzed in this study. This data can be found here: https://www.gtarsc.com.

## Author contributions

JZ, SW, and YC: conceptualization. JZ: software, data curation, and writing – original draft preparation. YT and YW: formal analysis. SW and YC: writing – review and editing and supervision. All authors have read and agreed to the published version of the manuscript.

## Conflict of interest

The authors declare that the research was conducted in the absence of any commercial or financial relationships that could be construed as a potential conflict of interest.

## Publisher’s note

All claims expressed in this article are solely those of the authors and do not necessarily represent those of their affiliated organizations, or those of the publisher, the editors and the reviewers. Any product that may be evaluated in this article, or claim that may be made by its manufacturer, is not guaranteed or endorsed by the publisher.

## Footnotes

^1^Shenyin Wanguo Industry Classification 2021 standard, China ‘s industry can be divided into agriculture, forestry, animal husbandry and fishery, basic chemical industry, steel, non-ferrous metals, electronics, automobiles, household appliances, food and beverage, textile and apparel, light industry manufacturing, pharmaceutical biology, public relations, transportation, real estate, commercial retail, social services, banking, non-bank finance, comprehensive, building materials, building decoration, power equipment, mechanical equipment, national defense and military industry, computer, media, communication, coal, petroleum and petrochemical, environmental protection and beauty care.
